# The Development of Generalized Motor Program in Constant and Variable Practice Conditions

**DOI:** 10.3389/fpsyg.2019.02760

**Published:** 2019-12-11

**Authors:** Stanisław H. Czyż, Martin Zvonař, Elric Pretorius

**Affiliations:** ^1^Department of Sport Didactics, University School of Physical Education in Wrocław, Wrocław, Poland; ^2^Faculty of Sports Studies, Masaryk University, Brno, Czechia; ^3^Physical Activity, Sport and Recreation Research Focus Area, North-West University, Potchefstroom, South Africa

**Keywords:** practice conditions, variability of practice, specificity of practice, especial skill, generalized motor program, motor learning

## Abstract

The main objective of our study was to determine whether constant and variable practice conditions lead to the development of different memory representations (GMP) and as a result, they benefit performance of a skill differently. We compared one of the Generalized Motor Program (GMP) invariant features, i.e., relative timing, of the same variation of skill developed in constant and variable practice conditions. In two experiments, participants, naïve to the basketball, were practicing free throws, receiving the same amount of practice. In constant conditions they practiced at one distance only (4.57 m), whereas in variable conditions they practiced at seven (2.74, 3.35, 3.96, 4.57, 5.18, 5.79, and 6.4 m) and five (3.35, 3.96, 4.57, 5.18, and 5.79 m) distances, in Experiments 1 and 2, respectively. We found that relative timing of skills developed in constant and variable practice conditions is the same, confirming that these practice conditions form the same memory representation. However, we also observed that constant practice (CP) conditions resulted in overall shorter movement time as compared to the skill practiced in variable conditions. We hypothesized that it may be due to the facilitation of parameters assignment as it takes place in especial skill.

## Introduction

Motor learning dynamic has been described by several models and theories. They share a common assumption that people acquire motor skills in a similar way, i.e., going through distinct stages (Magill and Anderson, 2017). One of such models, i.e., Gentile’s model of motor learning (Gentile, 1972, 2000) recognizes two levels: an initial stage and latter stages of learning. In the initial stage, a learner has to acquire a movement coordination pattern and has to learn to discriminate conditions that determine the movement characteristics from the one that does not influence it. In the latter stages of learning, a goal of learning differs depending on the type of skill. If a closed skill (Fitts and Posner, 1967) is to be learned then the goal is to fixate to movement characteristics, whereas if the open skill is learned the diversification of the movement pattern becomes the most important. Since open skills are performed in mutable and unpredictable situations, the movement characteristics of open skills have to be adaptable in order to satisfy the environmental requirements. On the other hand, closed skills require fixation of the movement characteristics, since the environmental context is stable. As a result, in the later stages of learning, these two skills require different practice conditions. Open skills are usually practiced in variable (VP) while closed skills in constant practice (CP) conditions.

Given there are different goals of learning open and closed skills, CP and VP benefit learning differently. CP benefits practiced variation of skill, giving it an advantage in performance over all other, non-practiced variations of a skill. Moreover, CP may eventually lead to the development of so-called especial skills (Keetch et al., 2005; Breslin et al., 2012b). Especial skill is usually defined as one variation of a skill which has a special status within other variations of skill and is distinguished by its enhanced performance capability relative to the other variations of a skill (Keetch et al., 2005). In contrast, VP better prepares for novel situations, i.e., it prepares better for the performance of a non- practiced variation of skill. It promotes transfer.

Although the benefits of CP and VP are well-known and well-evidenced (e.g., Van Rossum, 1990), there have been no previous attempts to explain what are the mechanisms differentiating benefits of CP and VP: why CP is better for closed and VP for open skills. One of the hypothesis may be analogous to what was proposed by Keetch et al. (2005) while speculating about the mechanisms underlying the emergence of especial skill. They hypothesized that the CP may form a separate new Generalized Motor Program (GMP) that optimizes the performance of a practiced variation of a skill, i.e., an especial skill, over non- practiced variations of a skill (Keetch et al., 2005). Although, the original hypothesis was falsified later (Breslin et al., 2010) it still may be advanced while considering the mechanisms differentiating benefits of CP and VP. There have been no previous attempts to test whether these different benefits of VP and CP may be due to the development of different GMPs, i.e., different GMP for a skill acquired in CP and different GMP acquired in VP. In order to test this hypothesis, we decided to develop one skill variation in CP and compare it to the same variation of skill but developed in VP. We assumed that if a learner has the same amount of practice as in the study by Breslin et al. (2012a) we should be able to recreate the especial skill effect in CP as well.

Given that movements executed by one GMP have to share, *inter alia* relative timing, one of the invariant features (Schmidt, 1975, 2003), we decided to look at the kinematic of the skill developed in CP and VP. This approach was used previously by Breslin et al. (2010). If the relative timing was different in the same variation of skill developed in VP as compared to CP, then we could confirm our hypothesis, saying different practice conditions lead to different GMPs. However, if the relative timing in both conditions was the same, we could say that both practice conditions develop same GMPs and different benefits these practice conditions bring, are not related to different memory representations.

The primary objective was to determine whether CP conditions develop different GMP as opposed to the variable practice conditions. We applied the procedure of Breslin et al. (2012a). Given, the especial skill is a unique skill variation formed in CP, we assumed that the amount of the practice in our experiments should be at least at the level described by Breslin and colleagues. We could develop especial skill in CP and compare it to the same variation of the skill but developed in VP. We assumed that the characteristics of the especial skill (Keetch et al., 2005) could help us differentiating both skills. Therefore, our secondary objective was to replicate the results of the study by Breslin and colleagues, i.e., we wanted to determine whether limited practice in constant conditions leads to the development of an especial skill.

## Materials and Methods

The experimental design was followed. In two experiments, we manipulated the distance at which free throws are practiced. The permission to conduct the first experiment was granted by the Research Ethics Committee of the Faculty of Sport Studies at Masaryk University, Czechia. The permission to conduct the second experiment was particularly granted by the Health Research Ethics Committee of the Faculty of Health Sciences (NWU-00180-1 5-A1), at the North-West University, South Africa. Both experiments were conducted according to the Declaration of Helsinki. Participants signed informed consent before the commencement of the study and they could resign from the study at any time.

Our samples size was calculated (Statistica 13.3) based on average shooting accuracy (means and standard deviations) reported for pre- and posttest in CP condition group by Breslin et al. (2012a) in Table 1. We assumed that if the especial skills has to be present in novice basketball players, we need to obtain the difference between pretest and posttest as reported by Breslin and colleagues. We set up alpha = 0.05 and power goal = 0.8. The estimated sample size was seven participants per group.

**TABLE 1:** A percentage of overall movement time of five kinematic landmarks: peak velocity (A), peak flexion (B), peak acceleration (C), peak velocity (D), and negative peak acceleration (E). Standard errors (SE) for the whole cohort provided in brackets.

**Landmarks**						
		**A**	**B**	**C**	**D**	**E**
**Group**	**Test**	**(*SE* = 6.208)**	**(*SE* = 7.071)**	**(*SE* = 6.111)**	**(*SE* = 5.161)**	**(*SE* = 4.370)**
Variable practice group	Pretest	70.823	51.360	60.281	66.415	71.982
	Posttest	80.012	64.171	68.822	73.249	73.799
Constant practice group	Pretest	74.624	38.612	53.118	64.937	75.175
	Posttest	73.283	35.357	56.972	68.555	76.879

### Experiment 1

#### Method

##### Participants

Twenty participants were recruited, however, only sixteen were analyzed. Four participants were excluded from the analysis because they either did not show up during all practice sessions or/and the retention test or the quality of the kinematic recordings was not high enough for the analysis (markers were not detected). Eventually, sixteen participants (mean age: 23.6 ± 0.93 years) without prior experience in basketball were randomly assigned to two groups. “Prior experience” was defined as any organized (i.e., with trainer/coach/instructor etc.) training for more than 3 months, at least once a week, apart from activities during physical education classes or occasional, recreational play (without supervision). One participant from the CP group trained MMA, one soccer, two ice-hockey and one long run. One participant from the VP group trained American football, two participants swimming, one participant baseball, and three participants played soccer. The rest of the participants was not involved in any organized sport.

##### Procedure and apparatus

Both groups were practicing basketball free throws for five consecutive days, performing a total of 105 free throws per day. This number does not exceed the amount of practice (also during testing) received in previous studies (e.g., Keetch et al., 2005; Breslin et al., 2010). In the publication by Czyż et al. (2013), participants performed 175 shots per day. According to the experienced basketball trainers Stanislaw Czyz contacted before conducting the study in 2013, the number of shots in any basketball training, is similar or even greater. It applies to experienced and inexperienced basketball players. The CP group practiced throws only from a free throw distance (4.57 m from the board), whereas the VP group practiced at seven different distances: 2.74, 3.35, 3.96, 4.57, 5.18, 5.79, and 6.4 m fifteen throws per distance per day (105 throws/day in total). Shots were performed in a quasi-random order, i.e., no more than two shots per distance were taken in a row (Keetch et al., 2005) and were taken from behind markers placed on the floor (2 cm wide tape, 10 cm long).

The shot efficiency pretest and posttest were performed. Pretest, on the 1st day before shooting practice had started, and posttest on the last day of practice after last shooting training had finished. They consisted of 15 shots per seven distances. A percent of success was calculated. A successful shot was scored 1, whereas a miss was scored 0 (Keetch et al., 2005; Czyż et al., 2015). A quasi-random shooting was applied during tests and practice sessions, although, for a logistic reason, five recorded shots at the free-throw distance was done in blocked order.

All of the participants were familiar with basketball free throws, as basketball is a part of the physical education curriculum in Czechia schools. Participants were instructed to throw the ball like in the basketball free throws.

##### Kinematic analysis

During pretest and posttests, the first five shots at the distance 4.57 m were recorded using Simi Reality Motion Systems with eight cameras (Basler asA640-120gc, 100 Hz). A 7 Hz low pass filter with 2nd order low-pass was used and an optimal cut-off frequency based on the residual analysis was applied (Winter, 2009). The kinematic data gathered was used for further analysis (SIMI Motion software 9.0.5). The system had been calibrated statically and dynamically before recording started and was re-calibrated during testing if environmental factors changed (e.g., sunlight).

A total of nine reflective markers were placed on the skin on the dominant side of the participant: distal end of the fifth metatarsal of the toe, lateral malleolus of the ankle, lateral condyle of the femur and on the greater trochanter of the femur, distal end of the middle finger just below the nail, on the hand just below the middle finger, ulnar styloid of the wrist, lateral epicondyle of the elbow and on the acromion process of the shoulder. Participants wore sleeveless shirts or no shirts.

Since the greatest angular range of motion during the propulsion phase of the shot can be noticed in elbow (Button et al., 2003), we used elbow angle for analysis as it was used previously (Breslin et al., 2010).

Recordings, as well as practice sessions, took place on the university basketball court. The start of the throw was defined as the point at which the shooting arm’s elbow marker was at its lowest position during preparation (Lam et al., 2009), whereas the ending point as a point at which the hand’s marker was at its highest position following release of the ball.

##### Data analysis

**FIGURE 1:**
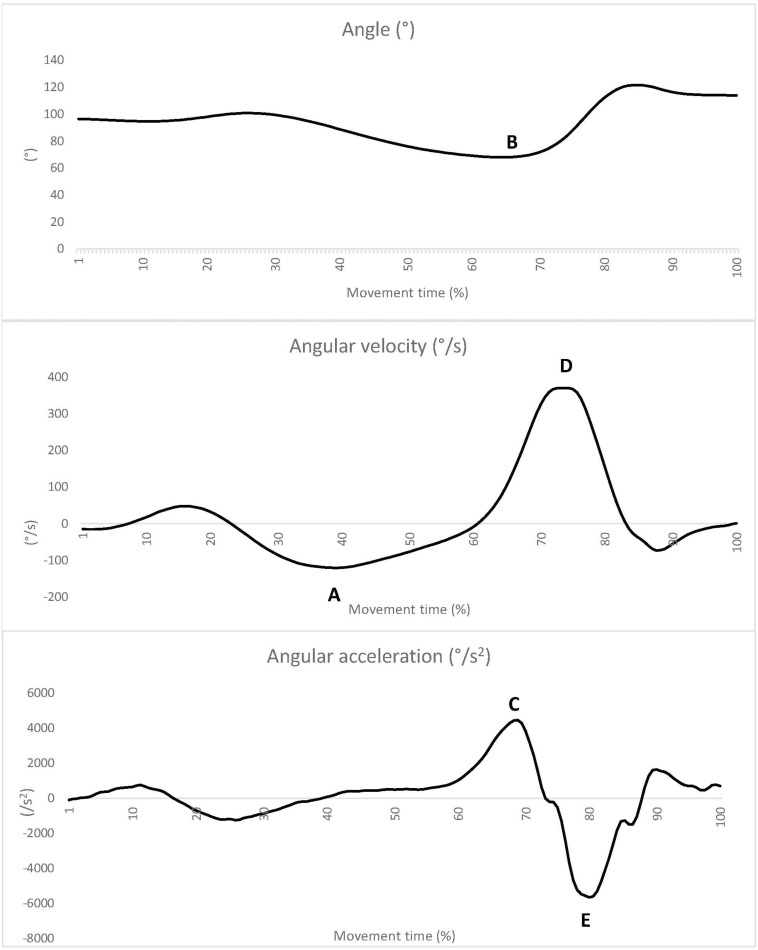
Landmarks A-E (peak velocity-A, peak flexion-B, peak acceleration-C, peak velocity-D and negative peak acceleration -E) on an exemplary graph representing one of the participants: A – angle, B – angular velocity, C – angular acceleration. The lines were smoothed using the moving average (based on 20 following results of the full data set, including the one representing particular time point).

The relative time as a percentage of overall movement time to reach five kinematic landmarks in the elbow joint was analyzed (Schneider and Schmidt, 1995; Breslin et al., 2010). These landmarks were: negative peak velocity (°/s) (A), peak flexion (°) (B), peak acceleration (°/s^2^) (C), peak velocity (°/s) (D), and negative peak acceleration (°/s^2^) (E) (Figure 1).

In a linear mixed model, a random intercept (participants treated as a random effect), test (pretest-posttest), group (CP – VP) and test^∗^group interaction were treated as fixed effects. Unstructured covariance matrix/structure was used. Bonferroni adjustments for multiple comparisons were applied. The variance/covariance of the 10 shots per participant (5 pre, 5 post) has been taken into account within the unstructured covariance matrix and in the calculation of the variance that was used in the effect size calculations.

We used a previously applied method to detect especial skills (Keetch et al., 2005, Experiment 1). Average percentage accuracy scores (based on a 2-point scoring system) were calculated for all the trials across each of the seven distances. We used these scores to compute regression lines for all of the shooting distances but the free-throw distance (4.57 m) for each participant. Based on regression equations, we estimated predicted shot efficiency at the distance of 4.57 m and compared predicted and real shot efficiency means at the 4.57 m distance using two-tailed paired *t*-tests.

#### Results

We calculated the relative time as a percentage of overall movement time to reach five kinematic landmarks. In the VP group, all kinematic landmarks occurred relatively later in posttest than in pretest (see Table 1 for mean values). However, kinematic landmarks A-D in posttest in CP group occurred relatively earlier than in VP group. Only landmark E in CP occurred later than in VP group (see Table 1 for details).

We computed a linear mixed model as described above. We did not find any significant main (i.e., test or group) or interactions (group^∗^test) effects while analyzing landmarks A and E (Table 2).

**TABLE 2:** Results of the linear mixed model for Experiment 1: *F*-values, *p*-values, and *ES* – effect sizes. Main effects (group: CP vs. VP; test: pretest vs. posttest) and interaction (group^∗^test).

		**Numerator**	**Denominator**			
**Landmark**	**Source**	***df***	***df***	***F***	***p***	***ES***
A	Intercept	1	14	363.519	0.000	
	Group	1	14	0.035	0.854	0.051
	Test	1	142	0.981	0.324	0.138
	Group^∗^test	1	142	1.767	0.186	
B	Intercept	1	14	94.170	0.000	
	Group	1	14	4.530	0.052	0.887
	Test	1	142	4.909	0.028	0.204
	Group^∗^test	1	142	13.874	0.000	
C	Intercept	1	14	201.983	0.000	
	Group	1	14	1.276	0.278	0.462
	Test	1	142	9.901	0.002	0.301
	Group^∗^test	1	142	1.415	0.236	
D	Intercept	1	14	379.205	0.000	
	Group	1	14	0.194	0.667	0.167
	Test	1	142	6.709	0.011	0.282
	Group^∗^test	1	142	0.635	0.427	
E	Intercept	1	14	677.095	0.000	
	Group	1	14	0.300	0.592	0.173
	Test	1	142	0.570	0.452	0.097
	Group^∗^test	1	142	0.001	0.981	

There were significant differences in relative timing of landmark B (see Table 2): pretest-posttest effect: *F*(1,142) = 4.9; *p* = 0.028; *ES* = 0.204. The group effect *F*(1,14) = 4.53; *p* = 0.052 was insignificant; and interaction test^∗^group was significant [*F*(1,142) = 13.87, *p* < 0.001]. Effect sizes for the interaction comparisons (landmark B) were small (VP group in pretest vs. posttest *ES* = 0.138), medium (CP group in pretest vs. posttest *ES* = 0.546; and CP vs. VP group in pretest *ES* = 0.544) and large (CP vs. VP group in posttest *ES* = 1.23).

We found significant difference of landmark C in pretest as compared to posttest results [*F*(1, 142) = 9.9, *p* = 0.002; *ES* = 0.301]. The group as well as interaction effects were not significant (*F* = 1.276, *p* = 0.278; *F* = 1.415, *p* = 0.236, respectively).

Similarly, we found significant difference landmark D in pretest as compared to posttest results [*F*(1, 142) = 6.7, *p* = 0.01, *ES* = 0.282]. The group as well as interaction effects were not significant (*F* = 1.194, *p* = 0.667; *F* = 0.635, *p* = 0.427, respectively).

##### Movement time

We used the same model as in the previous analysis for analyzing movement time (MT) (Table 3). The only significant effect was the group effect. The mean MT in the VP (*M*_VP_ = 1.039; *SE* = 0.088 s) was significantly longer than in CP (*M*_CP_ = 0.494, *SE* = 0.088). The effect size was large *ES* = 1.72.

**TABLE 3:** Results of the linear mixed model with the movement time (MT) as a dependent variable. *F*-value, *p*-value and *ES* – effect size.

**Source**	**Numerator *df***	**Denominator *df***	***F***	***p***	***ES***
Intercept	1	14	151.744	0.000	
Group	1	14	19.120	0.001	1.721
Time	1	142	1.160	0.283	0.110
Group^∗^Time	1	142	2.368	0.126	

We compared the MT in VP and CP in pretest and posttest (Table 4). We found that the MT in VP lengthened in posttest as compared to the pretest. The reverse effect was observed in CP group – MT in posttest was shorter than in pretest.

**TABLE 4:** Mean movement time (MT) in CP and VP in pretest and posttest. *ES* – effect size. The interaction effect was insignificant (*p* = 0.126), therefore no *post hoc* analysis was performed.

	**Pretest**	**Posttest**	***ES***
CP	0.537	0.452	0.268
VP	1.031	1.046	0.047
*ES*	1.563	1.879	

##### Especial skill

We did not notice any especial skill effects in VP group in pretest (mean real shot efficiency = 33.333 ± 17.457; mean predicted shot efficiency = 28.471 ± 8.227; *t*(7) = 1.138, *p* = 0.292) or posttest (mean real shot efficiency = 27.5 ± 12.051; mean predicted shot efficiency = 27.36 ± 11.408; *t*(7) = 0.046, *p* = 0.964). The especial skill was also absent in CP group in pretest (mean real shot efficiency = 20.833 ± 7.506; mean predicted shot efficiency = 26.527 ± 10.941; *t*(7) = −1.245, *p* = 0.253) or posttest (mean real shot efficiency = 27.5 ± 13.062; mean predicted shot efficiency = 27.638 ± 8.217; *t*(7) = −0.024, *p* = 0.9812).

#### Discussion

We had two objectives in this study: to determine whether CP conditions develops different GMP as opposed to the variable practice conditions. Secondly, we expected an especial skill to emerge in a limited number of trials in CP conditions.

In regards to the latter objective, we were unsuccessful at developing an especial skill in CP. Although our participants performed more shots during practice than participants in Breslin et al. (2012a) study, accumulating 525 shots in total as compared to 300 in their study, we did not notice an especial skill effect in the posttest. We may speculate that because we used a different method of scoring, i.e., a 2-point system (as Keetch et al., 2005) not the 4-point as Breslin et al. (2012a) did, we were unable to detect especial skills effect in our participants. Perhaps, the 4-point scoring system is more sensitive and therefore, we would have found an especial skill if we had used it.

We were not able to determine whether skills developed in CP are governed by a different GMP than a GMP developed in VP, either. We found no difference between CP and VP in posttests and the only significant interaction effect was found in landmark B. Given, that the only significant differences between landmarks were found between pre- and posttests results we could assume that CP and VP groups developed the same GMP governing shots at the distance 4.57 m. Unfortunately, the significant interaction effect found in landmark B blurred our results. As Schneider and Schmidt (1995) pointed out, relative timing is essentially invariant, should a unit of action be governed by a single GMP. This “*invariance is one of the important defining features of GMPs*” (Schneider and Schmidt, 1995, p. 44). Therefore, we cannot claim whether VP develops a different GMP than the CP but at the same time, we should be very cautious to claim otherwise.

The significant difference in MT, as revealed in the analysis, does not indicate whether CP and VP develop different GMPs. The only way to infer that the GMPs were different is to look at the temporal structure of the movement, i.e., look at the proportion of time needed to achieve defined landmarks. The amount of time spent on a shot is not an invariant feature *per se*. Although, what was interesting in the MT analysis, was the fact that VP significantly lengthened the MT in the posttest.

Considering the aforementioned shortcomings and results we decided to conduct another experiment, using different participants and the same task. We changed also testing conditions. Instead of a posttest as used previously (Breslin et al., 2012a), we decided to perform a 1-day retention test. As opposed to posttest, retention test assesses the learning better and gives us the idea of (relative) permanence or persistence of learning (Magill and Anderson, 2017). We decided to use a 4-point scoring system as used previously by Breslin et al. (2012a).

### Experiment 2

#### Method

##### Participants

Twenty healthy participants were randomly divided into two groups: variable practice group (VP1 – number one differs this group from the group in Experiment 1) and CP group (CP1). Ten participants (mean age = 22.3, SD = 2.24) were allocated to the VP1 group and ten participants (mean age = 21.3, SD = 1.16) to the CP1 group. All of them had no “prior experience” in basketball. “Prior experience” was defined as in Experiment 1. Five participants in VP1 trained rugby, one athletics, one hockey, one cricket, and one netball. In CP1, four participants trained rugby, one athletics, one tennis, one netball, one cricket, and one squash. One participant in CP1 and one participant in VP1 reported no previous experience in any sport.

##### Procedure and apparatus

Over five consecutive days in total, the first and the 5th day were dedicated to the pretest and retention tests, respectively, whereas days 2–4 (3 days in total) were designated to the acquisition phase [similarly as in Breslin et al. (2012a)]. Unlike in Experiment 1, and like in Breslin et al. (2012a), participants were tested at five distances: (3.35, 3.96, 4.57, 5.18, and 5.79 m). The shot efficiency was calculated based on a 4-point scoring system used in previous studies (Hardy and Parfitt, 1991; Keetch et al., 2005; Breslin et al., 2010, 2012a). Zero points were awarded for a miss, one point was awarded when the ball bounced off the rim but did not pass through the basket, two points were awarded when the ball passes the basket but it bounced before, and three points were awarded for a “swish” (the ball passed the basket without bouncing before). The percentage of the shot efficiency was calculated out of 60 points, i.e., number of maximum potential points in 20 shots in the pretest and, analogically, 20 shots in retention test (Keetch et al., 2005; Breslin et al., 2012a).

During the acquisition phase, CP1 participants performed 100 shots per day per one distance – free-throw distance (4.57 m). The VP1 participants performed 100 shots per day per five distances (3.35, 3.96, 4.57, 5.18, and 5.79 m), 20 shots per distance. All participants accumulated 300 shots in the acquisition phase (days 2–4) and 200 shots in pretest and retention tests. The informed consent forms and personal information questionnaires were collected before the testing commenced. The participant then had a five to 10-min warm-up period designated to stretching and preparing for the experiment. Shots were taken in a quasi-random order, with no more than two shots per distance in a row.

Participants were shown how to execute shots using two hands (from above their heads). The demonstration was done by the same person, the main investigator, who was familiar with basketball.

##### Kinematic analysis

We applied the same procedure as in Experiment 1, however, we used different recording equipment: the Qualisys Track Manager (QTM, Qualisys AB, Sweden) software and eight cameras (OQUS 3+, Qualisys AB, Sweden, resolution: 1280 × 1224 pixels, 200 Hz) located in a circular manner. Unlike in Experiment 1, tests and practice sessions took place in a laboratory where cameras and professional basketball board were mounted to the wall.

All shots were recorded with a digital high-speed camera (Exilim, Casio EX-ZR10, 40 fps) in order to score shots appropriately. The first five shots taken from the free-throw line (i.e., 4.57 m) during pretest and posttest were recorded for kinematic analysis.

We defined starting and ending points similarly to the Lam et al. (2009) procedure, i.e., a starting point of the movement as the point at which the shooting arm’s elbow marker was at its lowest position during preparation, whereas the ending point as the point 20 frames after the hand’s marker was at its highest position following release of the ball.

Unlike Experiment 1, all participants practiced and were tested barefoot.

##### Data analysis

Kinematic data analysis was done using the same procedure as in Experiment 1. Additionally, we analyzed inter-joint coordination for each throw for the shoulder–elbow and elbow–wrist joint pairs in the anterior-posterior (*y*) and vertical (*z*) axes using correlation coefficients (De Oliveira et al., 2007; Breslin et al., 2010). We applied the same linear mixed model with a random intercept (participants treated as a random effect), test (pretest-retention test), group (CP – VP) and test^∗^group interaction were treated as fixed effects as in previous analysis.

In order to detect especial skill, we applied the method used originally by Breslin et al. (2012a) while recreating the especial skill effect in CP. Average percentage accuracy scores (based on a 4-point scoring system) were calculated for all the trials across each of the five distances. We used these scores to compute regression lines for all of the shooting distances but the free-throw distance (4.57 m) for each participant. Based on regression equations, we estimated predicted shot efficiency at the distance of 4.57 m and compared predicted and real shot efficiency means at the 4.57 m distance using two-tailed paired *t*-tests.

#### Results

Likewise in Experiment 1, we calculated the relative time as a percentage of overall movement time to reach five landmarks (Table 5) and computed a linear mixed model with a random intercept (participants treated as a random effect), test (pretest-retention test), group (CP1 – VP1) and test^∗^group interaction (fixed effects). The mean values for each kinematic landmark are presented in Table 6.

**TABLE 5:** Results of the linear mixed model for Experiment 2: *F*-values, *p*-values, and ES – effect sizes. Main effects (group: CP1 vs. VP1; test: pretest vs. posttest) and interaction (group^∗^test).

		**Numerator**	**Denominator**			
**Landmark**	**Source**	***df***	***df***	***F***	***p***	***ES***
A	Intercept	1	18.025	3762.261	0.000	
	Group	1	18.025	1.496	0.237	0.250
	Test	1	177.269	2.891	0.091	0.226
	Group^∗^TEST	1	177.269	0.414	0.521	
B	Intercept	1	18.005	1649.871	0.000	
	Group	1	18.005	4.916	0.040	0.656
	Test	1	177.087	29.475	0.000	0.609
	Group^∗^test	1	177.087	17.755	0.000	
C	Intercept	1	18.082	2255.834	0.000	
	Group	1	18.082	4.043	0.059	0.381
	Test	1	177.376	9.472	0.002	0.417
	Group^∗^test	1	177.376	8.988	0.003	
D	Intercept	1	18.013	12500.113	0.000	
	Group	1	18.013	7.849	0.012	0.732
	Test	1	177.136	19.615	0.000	0.538
	Group^∗^test	1	177.136	15.177	0.000	
E	Intercept	1	17.902	5030.632	0.000	
	Group	1	17.902	0.764	0.394	0.169
	Test	1	177.183	5.547	0.020	0.318
	Group^∗^test	1	177.183	7.027	0.009	

**TABLE 6:** Results of the pairwise comparisons for landmarks A–E. Mean values of relative timing for constant (CP1) and variable (VP1) practice groups, *p*-values (*p*) and effect sizes (*ES*) provided in columns (comparison between CP1 and VP1 in pre- and retention test, accordingly) and rows (comparison between pre- and retention test results for CP1 and VP1, respectively).

	**Pretest**	**Retention test**	***p***	***ES***
**Landmark A**				
CP1	85.306	89.753	0.100	0.312
VP1	90.090	92.094	0.455	0.141
*p*	0.178	0.504		
*ES*	0.336	0.164		
**Landmark B**				
CP1	67.396	81.594	0.000	1.082
VP1	82.202	82.202	0.390	0.140
*p*	0.002	0.569		
*ES*	1.129	0.180		
**Landmark C**				
CP1	71.628	86.765	0.000	0.823
VP1	86.1	86.298	0.955	0.010
*p*	0.002	0.914		
*ES*	0.787	0.030		
**Landmark D**				
CP1	83.329	89.458	0.000	1.011
VP1	90.638	91.031	0.706	0.060
*p*	0.000	0.376		
*ES*	1.205	0.260		
**Landmark E**				
CP1	83.61	92.378	0.001	0.675
VP1	90.449	89.931	0.834	0.040
*p*	0.032	0.428		
*ES*	0.527	0.190		

We found significant test effect (pre- vs. retention test) and interaction effects in all landmarks but landmark A. We also found group significant effect (CP1 vs. VP1) in landmarks B and D.

We run additional pairwise comparisons for significant effects (see Table 6). We found that in most cases the interaction effect was due to the significant differences between CP1 and VP1 groups in the pretest (for all landmarks) as well as due to the significant differences between pretest-retention test results in CP1 (for all landmarks).

**FIGURE 2:**
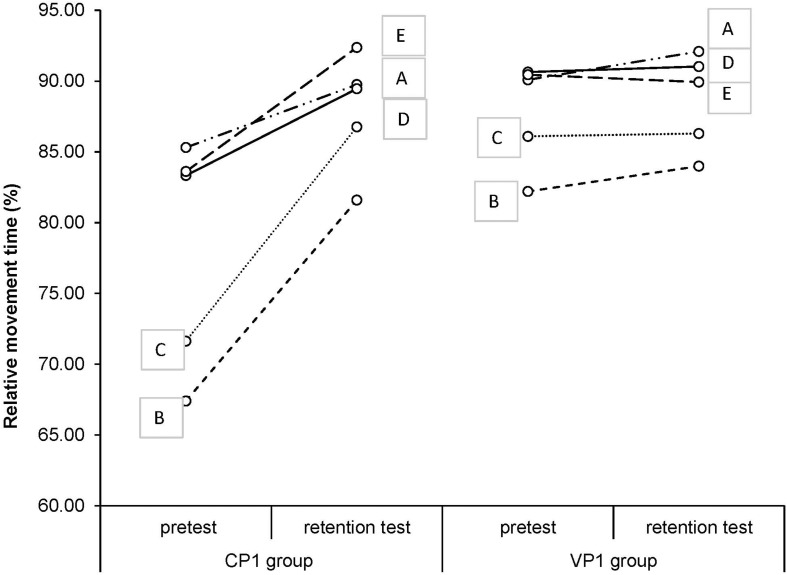
Relative movement time of all landmarks in pre- and retention test.

We did not find any significant differences between CP1 and VP1 in the retention test. The mean values of relative timing for all landmarks in pre- and retention test in CP1 and VP1 are visualized in Figure 2. As it can be noticed, the relative timing in CP1 and VP1 groups differed significantly in the pretest, but in the retention test, both groups had, noticeably, similar results.

**FIGURE 3:**
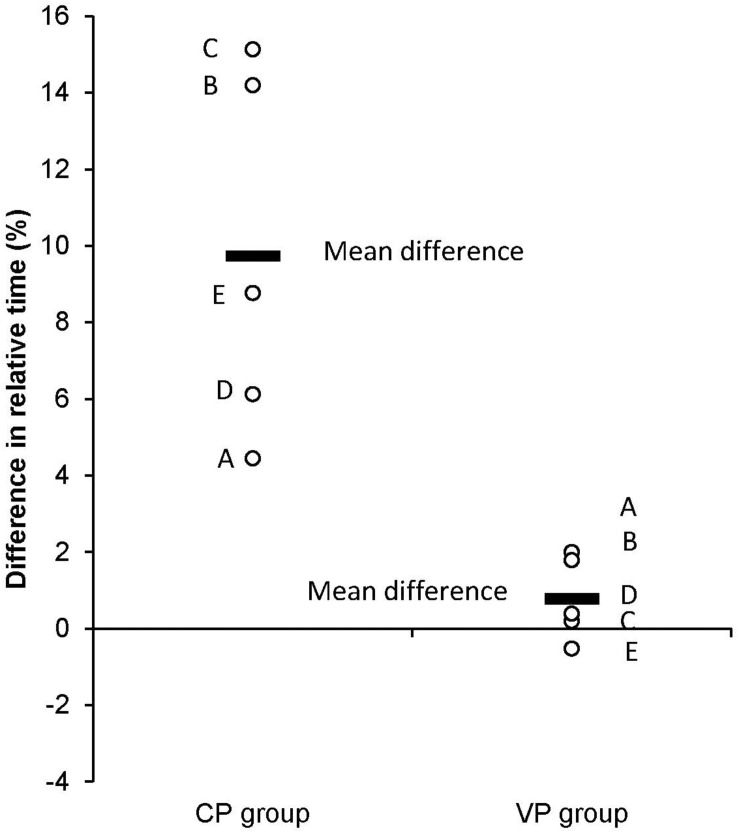
Differences in relative movement time between pre- and retention test results measured for CP1 and VP1 group. Percent points are used as measure units. The solid lines represent mean (for all landmarks) differences in relative timing between pre- and retention test for both groups.

On the other hand, in Figure 3, we showed the differences (percent points) between landmarks for CP1 and VP1 in pre- and retention test. The biggest difference was noticed in landmarks B and C in CP1 group, and it is reflected in big effect sizes (see Table 6). In VP1 group the differences between pretest and retention test relative timing were not bigger than 2% points (2% points for landmark A and 1.79 for landmark B – see Figure 3).

##### Movement time

Similarly to the analysis in Experiment 1, we looked at the movement time. We used the same linear mixed model to find any differences between CP1 and VP1 in pre- and retention test (Table 7).

**TABLE 7:** Degrees of freedom, *F*-and *p*-values and effect sizes (*ES*) for main effects (group: CP1 vs. VP1; test: pre- vs. retention test) and interaction.

	**Source**	**Numerator *df***	**Denominator *df***	***F***	***p***	***ES***
MT	Intercept	1	18.224	950.282	0.000	
	Group	1	18.224	6.446	0.020	0.614
	Test	1	178.321	3.174	0.077	0.223
	Group^∗^test	1	178.321	6.027	0.015	

Further, the pairwise comparison showed that the MT was not significantly different in the pretest (between CP1 and VP1) but it was in retention test (*p* = 0.002) and the effect size was big (0.922) (Table 8). Although, there were no significant differences between MT in pre- and retention test for CP1 group, similar trend as in Experiment 1 was found – the MT shortened in retention time as compared to the pretest. Again, similarly to the results in Experiment 1, MT in retention test in VP1 was longer in the retention test as compared to the pretest. This difference was significant and the effect size was moderate (0.531).

**TABLE 8:** Results of the pairwise comparisons for MT. Mean values of relative timing for constant (CP1) and variable (VP1) practice groups, *p*-values (*p*) and effect sizes (*ES*) provided in columns (comparison between CP1 and VP1 in pre- and retention test, accordingly) and rows (comparison between pre- and retention test results for CP1 and VP1, respectively).

	**Pretest**	**Retention test**	***p***	***ES***
CP1	2.025	1.976	0.635	0.084
VP1	2.204	2.515	0.003	0.531
*p*	0.270	0.002		
*ES*	0.306	0.922		

##### Inter-joint coordination

In order to determine whether coordination in CP1 and VP1 was characterized by a different pattern, we analyzed correlation coefficients for shoulder-elbow and elbow-wrist in the *z* and *y*-axis (see Table 9 for coefficients values). We examined the inter-joint coordination applying the same linear mixed model as previously but we did not find any significant main effects. We found only one interaction effect for shoulder – elbow coordination in axis *Z* [*F*(1,178 = 4.712, *p* = 0.031]. Further, the pairwise comparison revealed that the interaction effect was due to a significant difference between pre- and retention test results in CP1 (*p* = 0.008, *ES* = 0.349).

**TABLE 9:** Correlation coefficients and standard errors (*SE*) for each group (CP1 and VP1), axis (*Y* and *Z*) and test (pre- and retention).

	***Y***			***Z***		
		**Retention**			**Retention**	
	**Pretest**	**test**	***SE***	**Pretest**	**test**	***SE***
**Shoulder – Elbow**						
CP1	0.639	0.580	0.075	0.538	0.611	0.053
VP1	0.684	0.727		0.675	0.664	
**Elbow – wrist**						
CP1	0.415	0.473	0.111	0.960	0.986	0.018
VP1	0.498	0.426		0.939	0.970	

It can be noticed that correlations were moderate (elbow – wrist Y) and strong (shoulder – elbow and elbow – wrist X).

##### Especial skill

The especial skills effect was not present in either group, in pretest or retention test. Due to the fact, that predicted shot efficiencies at the 4.57 m distance were higher than real one, we cannot claim that one variation of skill (i.e., free throws at the 4.57 m distance) was outperformed, as compared to the other variations of a skill (free throws at other distances). In the VP1 group, the real shot efficiency at 4.57 m in a pretest was 36.5% (SD = 8.331) whereas the predicted was 45% (SD = 5.961). In retention test, the respective values were: real = 50% (SD = 8.571) and predicted = 50.541% (SD = 3.727). In CP1, the real shot efficiency in pretest was 40% (SD = 9.196), predicted mean = 47.642 (SD = 7.812). In retention test the mean real shot efficiency was = 44.5% (SD = 5.882) and predicted 50.083% (SD = 8.102).

#### Discussion

Our two objectives were the same as in Experiment 1, i.e., we wanted to determine whether practice in constant conditions may lead to the development of different GMP as opposed to the variable practice conditions. Secondly, we wanted to determine whether limited practice in constant conditions leads to the development of an especial skill. However, we changed testing and practice conditions as compared to Experiment 1. We used a 1-day retention test to detect especial skill in our participants and a 4-point scoring system (Hardy and Parfitt, 1991; Keetch et al., 2005; Breslin et al., 2010, 2012a). We also changed the practice conditions. Instead of 5 days of practice, our participants practiced for 3 days only, they were tested at five distances [as in Breslin et al. (2012a)], and the VP1 practiced at five distance (Breslin et al., 2012a).

Although we used a different scoring system, we did not detect an especial skill in CP1. It is difficult to say why we were unsuccessful. Perhaps, due to the differences in characteristics of the participants used in our experiments, or due to the previous experience participants had had, the amount of practice our participants received was too little to develop an especial skill. We cannot claim that using a different method of detecting especial skills, e.g., one of the two proposed by Czyż et al. (2013), would detect them since the real shot efficiency at 4.57 was lower than the predicted one.

Similarly to results in Experiment 1, we found significant effects in MT analysis: group effect (shorter MT in CP) and interaction effect (group^∗^test). The significant interaction effect was mostly due to the significant differences between CP1 and VP1 in the retention test as well as the significantly longer MT in the retention test as compared to the pretest in VP1.

The inter-joint correlation was strong and moderate and except for one correlation (shoulder – elbow in axis *Z*) they did not differ one from another. It means that the movements were quite consistent in both groups in pre- and retention tests.

It can be noticed in relative timing analysis that we probably had recruited two different groups of participants. They differed significantly in terms of the landmarks’ relative timing in the pretest (see Figure 2). However, what is even more interesting, there were no differences between CP1 and VP1 in retention testes. It means that our two groups with different (or no) movement patterns at the beginning were developing the same GMPs, although they practiced in different practice conditions.

## General Discussion

The primary objective of this study was to determine whether practice in constant conditions leads to the development of different GMP as opposed to the variable practice conditions. The secondary objective was to determine whether limited practice in constant conditions leads to the development of especial skill, i.e., replicate the results of Breslin et al. (2012a).

We found that a skill developed in CP and VP have the same relative timing in retention test (Experiment 2). The relative timing in posttest (Experiment 1) was almost the same, however, the results were not so clear – one landmark (B) significantly differed in terms of an interaction effect, group^∗^test interaction. The difference was mostly due to the differences found between pretest-posttest results. The effect of group (CP vs. VP results) was not significant. Considering our findings in both experiments, specifically, results obtained in Experiment 2, in which two groups (CP1 vs. VP1) significantly differed one from another in pretest but they did not in retention test, we may claim that regardless of the practice conditions, variable and CP develop same GMP.

We did not succeed at recreating an especial skill effect in either of our experiments. It was particularly surprising in Experiment 1, in which our participants received a bigger amount of practice (525 shots accumulated during practice sessions) as compared to that received by Breslin et al. (2012a) experiment (300 shots in total during practice session). We may speculate, that participants in Breslin et al. (2012a) study had more experience in basketball than ours, for example, playing more basketball at schools or in their leisure time. Of course, it could be otherwise – participants in our experiments may have had more experience in basketball itself or in a task similar to the basketball free-throw shooting. Whatever is true, we may assume that our participants differed from experienced ones. In another study by Breslin et al. (2010), the inter-joint coordination correlation coefficients in experienced basketball players were different than those found in ours. Breslin and colleagues found that correlation coefficients at the distance 4.57 m (15 ft) in the shoulder – elbow and elbow wrist joints in the vertical direction (*z*-axis) were above 0.9. whereas in our participants (in retention test), these coefficients were around 0.6 and above 0.95, accordingly. These differences were even higher when comparing anterior-posterior direction (*y*-axis). In Breslin et al. (2010) participants correlation coefficients were small (it was 0.28) in the shoulder – elbow joint and there was almost no correlation in elbow – wrist joint (correlation coefficient 0.04). In our participants (see Table 9) these correlations were medium (above 0.4 for elbow – wrist joint) and large in the shoulder – elbow joint (above 0.7 in VP1). Our finding may be explained by Bernstein’s theory of motor coordination (Bernstein, 1967), i.e., the inter-joint correlation (couplings) is higher at the initial phase of motor learning. This phase is called freezing (Savelsbergh et al., 2004). On the other hand, Breslin et al. (2010) participants were much more experienced (“with at least 10 years of experience”) and it may be assumed that they were in phase called exploiting or at least freeing as the inter-joint and information – movement coupling is different at these phases (Savelsbergh and Van Der Kamp, 2000).

Of course, there may have been many other reasons why we did not recreate an especial skill effect in our participants. However, we believe that the failure in the replication of Breslin et al. (2012a) procedure is an added value of this study (Zwaan et al., 2018). It is worth to mention that in another recently published study, we did not succeed at the recreation of especial skill effect either (Czyż et al., 2019).

### Movement Time

We consider our findings regarding changes and differences in movement time as very interesting. In Experiment 1, participants practicing in variable practice had significantly longer movement time than CP participants. A similar trend was found in Experiment 2. The CP1 groups shortened their MT due to the nature of their practice, whereas VP1 lengthen the MT. Why MT in CP was shorter than in VP? In previous studies on especial skill (Keetch et al., 2005; Simons et al., 2009; Breslin et al., 2010; Nabavinik et al., 2018) it was hypothesized, that parameterization may be the main cause for the emergence of especial skill. Practice in constant conditions may facilitate parameter assignment (Keetch et al., 2005), at least for the especial skill. If it is true, then parameters assignment in CP should be shorter than in VP. It could result in shorter MT, however, the memory representation (GMP) could be the same. This finding is in line with Lee et al. (2016) conclusions. They found that response time delay and motor program parameters appear to stem from two distinct processes. As the uncertainty and external perturbation increase, the response time and cost (loss function) associated with the motor programing effort increases too. Since VP groups in our experiments were practicing and tested in a quasi-random order, they experienced time and distance perturbation (participants were had been told to what distance they should go next just before they the changed). Hence it may be that the same motor program may be used in CP and VP and only timing and force parameters may be adjusted affecting MT as a result. This speculation should be tested in further research, as we compared only practiced variation of a skill. It would be interesting to test non-practiced variations of a skill practiced in constant and variable conditions.

Another possible explanation may be associated with the force × variability assumption (Schmidt et al., 1978). When the distance to the target increases, increased levels of force must be generated by an individual (Schmidt et al., 1979). As a result, increased levels of variability in force output is observed (Schmidt et al., 1979). On the other hand, variability in force output is associated with fatigue (Hunter et al., 2004). Therefore, one could speculate, that VP groups were more fatigued than CP and their MT was longer. This issue could be addressed in the next study.

The results of our study should be deliberated against its limits. First of all, our findings should be replicated in other studies (Zwaan et al., 2018). Second, especial skill should be developed in order to compare relative timing and MT of especial skill and the same variation of the skill but practiced in variable conditions. Third, other invariant features (Schmidt, 2003) and other skills could be considered in order to determine if the same GMP is developed in CP and VP.

The strength of our study is that, to the best of our knowledge, it is the first study looking at the mechanisms differentiating benefits of variable and CP conditions. We found that both practice conditions developed the same memory representation (GMP). We also found that different practice conditions affect movement time, however, the reason why should be furthermore investigated.

## Data Availability Statement

The datasets generated for this study are available on request to the corresponding author.

## Ethics Statement

The studies involving human participants were reviewed and approved by the Research Ethics Committee of the Faculty of Sport Studies at Masaryk University, Czechia and the Health Research Ethics Committee of the Faculty of Health Sciences (NWU-00180-1 5-A1) at the North-West University, South Africa. The patients/participants provided their written informed consent to participate in this study.

## Author Contributions

SC has contributed to the conception and design of the work, data collection in both experiments, performed statistical analyses, and drafted the manuscript. MZ contributed to the work by collecting the data in Experiment 1 and revising the work critically. EP contributed to the work by collecting the data in Experiment 2 and revising the work critically. All authors read and approved the final version of the manuscript.

## Conflict of Interest

The authors declare that the research was conducted in the absence of any commercial or financial relationships that could be construed as a potential conflict of interest.
